# Metabolic cell death in cancer: ferroptosis, cuproptosis, disulfidptosis, and beyond

**DOI:** 10.1093/procel/pwae003

**Published:** 2024-03-01

**Authors:** Chao Mao, Min Wang, Li Zhuang, Boyi Gan

**Affiliations:** Department of Experimental Radiation Oncology, The University of Texas MD Anderson Cancer Center, Houston, TX 77030, USA; Department of Molecular and Cellular Biology, Baylor College of Medicine, Houston, TX 77030, USA; Department of Experimental Radiation Oncology, The University of Texas MD Anderson Cancer Center, Houston, TX 77030, USA; Department of Experimental Radiation Oncology, The University of Texas MD Anderson Cancer Center, Houston, TX 77030, USA; The University of Texas MD Anderson UTHealth Graduate School of Biomedical Sciences, Houston, TX 77030, USA

**Keywords:** cell death, cancer treatment, ferroptosis, cuproptosis, disulfidptosis

## Abstract

Cell death resistance represents a hallmark of cancer. Recent studies have identified metabolic cell death as unique forms of regulated cell death resulting from an imbalance in the cellular metabolism. This review discusses the mechanisms of metabolic cell death—ferroptosis, cuproptosis, disulfidptosis, lysozincrosis, and alkaliptosis—and explores their potential in cancer therapy. Our review underscores the complexity of the metabolic cell death pathways and offers insights into innovative therapeutic avenues for cancer treatment.

## Introduction

Cell death plays a critical role in maintaining homeostasis within multicellular organisms. However, this delicate balance can be disrupted in various diseases, including cancer (Hotchkiss et al., 2009). Cell death can be broadly categorized into two types: nonregulated (accidental) cell death and regulated cell death. Nonregulated cell death occurs passively as a result of external factors such as physical or chemical damage and lacks the clear involvement of internal signaling and execution mechanisms. In contrast, regulated cell death is a highly controlled and orderly process orchestrated by a series of intracellular signaling and execution mechanisms (Bedoui et al., 2020; Galluzzi et al., 2018; Tang et al., 2019).

Recent studies have identified several unique forms of regulated cell death that arise from the overload or depletion of certain nutrients (e.g., glucose and amino acids) or metals (e.g., iron and copper) and resulting cellular metabolism imbalances. This form of cell death—metabolic cell death—has often been referred to as “cell sabotage” (Green and Victor, 2012). In contrast, “cell suicide” designates another category of regulated cell death directly triggered by signaling cascades and cell death executioner proteins that eliminate unwanted cells in organisms (Green and Victor, 2012). Apoptosis and ferroptosis are prime examples of cell suicide and cell sabotage, respectively. The distinguishing feature between these two categories of regulated cell death may be due to their developmental roles: cell suicide programs have clear developmental functions, such as sculpting the digits during development through apoptosis. However, whether cell sabotage programs have positive developmental roles remains somewhat unclear or subject to debate (Green and Victor, 2012).

Despite lacking clear developmental functions, a deeper understanding of the intricate mechanisms underlying metabolic cell death holds significant potential in shedding light on disease progression and paving the way for innovative therapeutic approaches in the treatment of various diseases, particularly cancer. Cancer cells frequently undergo metabolic reprogramming, revealing distinct metabolic vulnerabilities that can be targeted therapeutically by inducing specific metabolic cell death (Pavlova et al., 2022). This approach offers novel strategies to combat resistance to conventional therapies primarily designed to induce apoptosis in cancer cells (Carneiro and El-Deiry, 2020).

In this review, we examine the intricate mechanism of metabolic cell death and explore several recently discovered pathways, including the ferroptosis, cuproptosis, disulfidptosis, lysozincrosis, and alkaliptosis pathways. We have focused on ferroptosis, given the extensive literature regarding this mode of cell death. We also investigate the potential of targeting these specific cell death mechanisms in cancer therapy. A discussion of cell suicide programs and other conventional cell death pathways such as apoptosis, necroptosis, pyroptosis, and autophagic cell death is beyond the scope of this review, as is a comprehensive examination of all recently identified cell death pathways. For readers interested in these areas, several outstanding recent reviews providing in-depth insights into these topics are available (Bedoui et al., 2020; Galluzzi et al., 2018; Hadian and Stockwell, 2023; Koren and Fuchs, 2021; Leak and Dixon, 2023; Tang et al., 2019).

## Ferroptosis

The term “ferroptosis” was coined in 2012 to describe a distinctive form of iron-dependent cell death triggered by the abnormal accumulation of lipid peroxides on cellular membranes (Dixon et al., 2012). It stands apart from other types of regulated cell death, both morphologically and mechanistically (Chen et al., 2021c; Dixon et al., 2012). For example, ferroptotic cells do not exhibit typical apoptotic features such as chromatin condensation or caspase-3 cleavage (Tang et al., 2019) but instead display shrunken mitochondria with reduced mitochondrial cristae (although the precise mechanisms driving mitochondrial morphological changes during ferroptosis remain elusive) (Dixon et al., 2012). Another defining feature of ferroptosis is its blockade by iron chelators or lipophilic antioxidants, but not by inhibitors of other common cell death pathways (Dixon et al., 2012).

**Figure 1.**
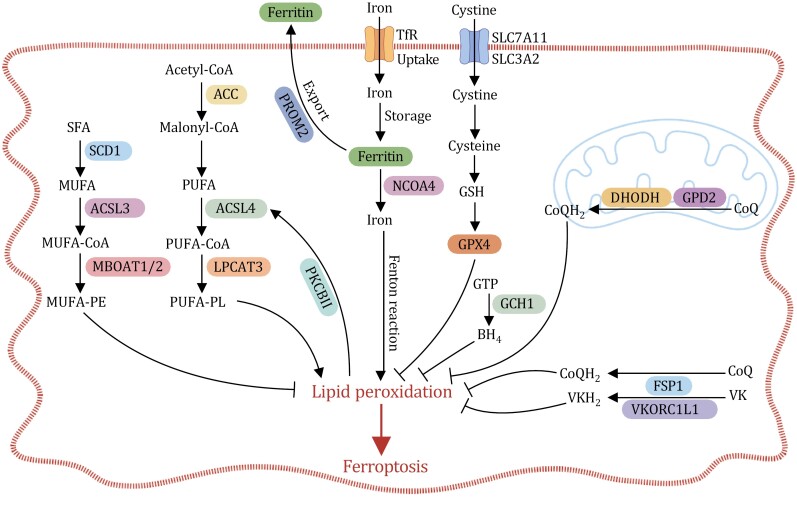
**Key mechanisms of ferroptosis.** Ferroptosis is caused by excessive lipid peroxidation, which mainly occurs on PUFA-PLs. PUFA-PL synthesis is regulated by ACSL4, LPCAT3, and ACC. MUFA-PEs, on the other hand, suppresses lipid peroxidation. MUFA-PE synthesis is regulated by SCD1, ACSL3, and MBOAT1/2. Ferroptosis is triggered by the iron-mediated Fenton reaction. Therefore, the sensitivity of ferroptosis is closely related to iron metabolism, involving processes such as iron uptake (e.g., via transferrin), storage (e.g., via ferritin), release (e.g., via NCOA4), and export (e.g., via PROM2). The GPX4-dependent and GPX4-independent (including CoQH_2_, VKH_2_, and BH_4_) pathways constitute the main antioxidant system to counteract lipid peroxidation and defend against ferroptosis. They work by inhibiting lipid peroxidation, thus preventing ferroptosis.

From a metabolic standpoint, ferroptosis signifies a cellular state in which the metabolic activities that fuel lipid peroxidation substantially surpass the buffering capacity of the cellular defense systems designed to counteract this process (Jiang et al., 2021). In this section, we will delve into lipid synthesis and peroxidation, as well as iron metabolism (Fig. 1), as key ferroptosis-driving mechanisms. We will then explore both glutathione peroxidase 4 (GPX4)-dependent and GPX4-independent defense mechanisms that counteract lipid peroxidation (Fig. 1). Finally, we will investigate potential strategies for engaging ferroptosis in cancer therapy.

### Lipid synthesis and peroxidation

Polyunsaturated fatty acids (PUFAs) are fatty acids that contain more than one double bond. The cellular membrane of mammalian cells is enriched with PUFA-containing phospholipids (PUFA-PLs) (Harayama and Riezman, 2018), and although this characteristic enhances the cellular membrane’s fluidity and facilitates signaling (Harayama and Shimizu, 2020), it comes at a cost. Unlike saturated fatty acids or monounsaturated fatty acids (MUFAs)—fatty acids containing no or one double bond, respectively—PUFAs are highly susceptible to peroxidation because they contain bis-allylic moieties. As a result, cells with a high PUFA content are particularly vulnerable to ferroptosis (Jiang et al., 2021).

During ferroptosis, lipid peroxidation primarily targets PUFAs already incorporated into PLs rather than free PUFAs (Jiang et al., 2021). Thus, the enzymes involved in PUFA-PL biosynthesis play a critical role in lipid peroxidation and ferroptosis. Acyl-CoA synthetase long-chain family member 4 (ACSL4) facilitates the ligation of free PUFAs to CoA to form PUFA-CoAs, which are subsequently re-esterified and integrated into PLs by lysophosphatidylcholine acyltransferase 3 (LPCAT3) to generate PUFA-PLs (Liang et al., 2022) (Fig. 1). Moreover, certain PUFAs, such as linoleic acid, cannot be synthesized *de novo* in mammalian cells and must be acquired from the diet or media (Lee et al., 2016). Linoleic acid can be converted to other PUFAs, such as arachidonic acid, through additional desaturation and elongation steps. The elongation reaction requires a supply of malonyl-CoA, produced by the acetyl-CoA carboxylase (ACC)-mediated carboxylation of acetyl-CoA (Lee et al., 2016). Consequently, the inactivation of ACSL4, LPCAT3, or ACC suppresses PUFA-PL synthesis and confers resistance to ferroptosis in cells (Dixon et al., 2015; Doll et al., 2017; Lee et al., 2020; Reed et al., 2022; Yuan et al., 2016). Another recent discovery revealed a positive feedforward loop facilitated by the protein kinase C beta (PKCβΙΙ)-ACSL4 signaling axis, which detects and amplifies lipid peroxides, thereby contributing to the execution of ferroptosis (Zhang et al., 2022) (Fig. 1). It is noteworthy that ACSL4 appears to play a differential role in ferroptosis triggered by different ferroptosis inducers (FINs) (Magtanong et al., 2022), although the underlying mechanisms remain unclear.

MUFAs, such as oleic acid and palmitoleic acid, are less prone to peroxidation than PUFAs because they lack bis-allylic moieties (Yang et al., 2016). In contrast to supplementing cells with PUFAs, supplementing cells with MUFAs efficiently suppresses lipid peroxidation and ferroptosis, likely by displacing PUFAs from PLs in the cellular membrane (Magtanong et al., 2019). As a result, the inactivation of the enzymes involved in MUFA-PL synthesis, such as stearoyl-CoA desaturase 1 (SCD1) and acyl-CoA synthetase long-chain family member 3 (ACSL3), sensitizes cancer cells to ferroptosis (Luis et al., 2021; Magtanong et al., 2019). Additional studies have revealed that membrane-bound O-acyltransferase domain-containing 1/2 (MBOAT1/2) selectively transfer MUFAs to lyso-phosphatidylethanolamine (lyso-PE), thereby inhibiting ferroptosis by increasing MUFA-PE levels and concurrently reducing PUFA-PE levels in cancer cells (Liang et al., 2023) (Fig. 1). These findings shed new light on the intricate interplay between MUFAs and PUFAs in regulating ferroptosis.

The peroxidation of PL-PUFAs is primarily instigated by the nonenzymatic Fenton reaction. This reaction leads to the formation of phospholipid radicals, which subsequently interact with other PUFAs, triggering a chain reaction and propagating lipid peroxidation (Liang et al., 2022). Enzymatic reactions mediated by arachidonate lipoxygenase (ALOX) and cytochrome P450 oxidoreductase (POR) have also been demonstrated to participate in lipid peroxidation (Yan et al., 2021; Yang et al., 2016; Zou et al., 2020); however, ALOX’s involvement in lipid peroxidation has been challenged by some studies (Shah et al., 2018; Zou et al., 2020), and the role of POR in promoting lipid peroxidation seems to be somewhat indirect and is primarily attributed to the generation of hydrogen peroxide (Yan et al., 2021).

### Iron metabolism

The iron-dependent nature of ferroptosis highlights the indispensable role of iron in facilitating the Fenton reaction, a driving force for lipid peroxidation (Conrad and Pratt, 2019). Furthermore, the significance of iron in ferroptosis extends beyond its catalytic involvement in the Fenton reaction; it also serves as a key cofactor for enzymes participating in lipid peroxidation, including ALOXs and POR.

Cellular iron homeostasis is tightly governed through the intricate coordination of the uptake, storage, use, and export processes for iron (Vogt et al., 2021) (Fig. 1). As a result, any disturbance in this finely tuned regulation can affect the level of the intracellular labile iron pool and thereby influence the sensitivity of cancer cells to ferroptosis (Chen et al., 2020). For instance, iron uptake facilitated by transferrin, lactotransferrin, and transferrin receptor (TfR) stimulates ferroptosis, with the transferrin receptor itself being identified as a ferroptosis marker (Chen et al., 2020; Feng et al., 2020). Intracellular labile iron, known for its high reactivity, is stored in ferritin. The degradation of ferritin via nuclear receptor coactivator 4 (NCOA4)-mediated ferritinophagy frees iron into the labile iron pool, effectively promoting ferroptosis (Gao et al., 2016; Hou et al., 2016). Conversely, prominin 2 exerts an inhibitory effect on ferroptosis by enhancing the export of iron (Brown et al., 2019). This intricate network of iron-related mechanisms collectively influences cellular sensitivity to ferroptosis, underscoring the multifaceted role of iron as both a catalyst and a regulator in this form of regulated cell death.

### GPX4-dependent defense mechanisms against ferroptosis

A multitude of defense mechanisms have evolved to thwart the excessive buildup of lipid peroxides on cellular membranes, thereby impeding the initiation of ferroptosis. These protective strategies can be broadly classified into GPX4-dependent and GPX4-independent systems.

The GPX4-dependent system, widely regarded as a cornerstone in defense against ferroptosis, hinges on the uptake of extracellular cystine through the system X_c_^−^ transporter. System X_c_^−^ functions as a glutamate/cystine antiporter and consists of the catalytic subunit solute carrier family 7 member 11 (SLC7A11, also called xCT) and the regulatory subunit solute carrier family 3 member 2 (SLC3A2, also named 4F2hc or CD98; it should be noted that SLC3A2 also associates with other amino acid transporters and participates in the transport of multiple amino acids. Given the pleiotropic nature of SLC3A2 in nutrient transport, this review predominantly centers on SLC7A11) (Bannai, 1986; Koppula et al., 2018; Nakamura et al., 1999; Sato et al., 1999) (Fig. 1). SLC7A11, which is frequently upregulated in cancer, acts as a key regulatory hub within the ferroptosis pathways (Koppula et al., 2021b). Its expression is governed by an array of proteins such as tumor protein p53 (TP53, also commonly known as p53), nuclear factor erythroid 2-related factor 2 (NFE2L2, also named NRF2), BRCA1-associated protein 1 (BAP1), yes-associated protein 1(YAP1)/WW-domain-containing transcription regulator 1 (WWTR1; also known as TAZ), and activating transcription factor 4 (ATF4) through transcriptional control; in addition, its levels can be regulated via posttranscriptional mechanisms that involve other proteins such as beclin 1, OTU deubiquitinase (OTUB1), and OTU deubiquitinase 5 (OTUD5 or DUBA) (Chen et al., 2017; Gao et al., 2021; Jiang et al., 2015; Liu et al., 2019; Sasaki et al., 2002; Sato et al., 2004; Song et al., 2018a; Wang et al., 2023b; Zhang et al., 2018). Furthermore, multiple compounds, such as erastin, sorafenib, and sulfasalazine, inhibit SLC7A11-mediated cystine transport. The inhibition of this transport by these compounds is associated with their ability to instigate ferroptosis in cancer cells (Dixon et al., 2012, 2014; Koppula et al., 2021b).

Intracellular cystine imported by SLC7A11 is subsequently reduced to cysteine. Cysteine, in turn, serves as the essential precursor for the synthesis of glutathione (GSH), a tripeptide of paramount importance in maintaining redox homeostasis (Lushchak, 2012). GSH is harnessed by GPX4 to catalyze the reduction of harmful lipid hydroperoxides into harmless lipid alcohols, and thus it effectively suppresses ferroptosis (Ursini and Maiorino, 2020; Weaver and Skouta, 2022) (Fig. 1). Pharmacological inhibition (e.g., by RSL3 or ML162 treatment) or the genetic ablation of GPX4 induces ferroptosis in a multitude of cancer cell lines (Jiang et al., 2021; Yang et al., 2014). Moreover, the constitutive or conditional deletion of *Gpx4* causes embryonic lethality or organ impairment in adult mice, presumably stemming from the *in vivo* induction of ferroptosis (Friedmann Angeli et al., 2014; Yant et al., 2003).

Recent research has provided novel insights into the regulatory mechanisms of GPX4. As a selenoprotein, GPX4 depends on the presence of its essential selenocysteine residue for its functional efficacy in countering ferroptotic processes (Ingold et al., 2018). LDL receptor-related protein 8 (LRP8)-mediated selenocysteine uptake has emerged as a critical mechanism in bolstering GPX4 protein synthesis and ferroptosis suppression (Alborzinia et al., 2023; Li et al., 2022). Other studies have underscored selenocysteine’s capacity to upregulate GPX4 levels through the modulation of transcriptional control mechanisms (Alim et al., 2019). In addition, cysteine not only provides the key precursor for synthesizing GSH but also stimulates GPX4 protein synthesis by activating mechanistic/mammalian target of rapamycin complex 1 (mTORC1) (Zhang et al., 2021). These multifaceted interplays among selenocysteine, cysteine, and GPX4 emphasize the importance of their partnership in safeguarding cells from ferroptotic damage. Adding yet another layer of complexity to the GPX4 regulatory landscape, a recent study determined that creatine kinase B (CKB) stabilizes GPX4 through phosphorylation, thereby enhancing its ferroptosis-suppressing capabilities (Wu et al., 2023). These studies have together unraveled the multifaceted pathways governing GPX4’s expression and activity in cells’ defense mechanisms against ferroptosis.

### GPX4-independent defense mechanisms against ferroptosis

The GPX4-independent defense system against ferroptosis involves a diverse set of radical-trapping antioxidants (RTAs) endowed with powerful ferroptosis-suppressing capabilities, such as ubiquinol (CoQH_2_), tetrahydrobiopterin (BH_4_), and reduced forms of vitamin K (Fig. 1). This intricate defense mechanism relies on specialized enzymes that orchestrate the production of these RTAs, including ferroptosis suppressor protein 1 (FSP1; previously known as AIFM2), dihydroorotate dehydrogenase (DHODH), glycerol-3-phosphate dehydrogenase 2 (GPD2), GTP cyclohydrolase 1 (GCH1), and vitamin K epoxide reductase complex subunit 1-like 1 (VKORC1L1) (Bersuker et al., 2019; Doll et al., 2019; Kraft et al., 2020; Mao et al., 2021; Soula et al., 2020; Wu et al., 2022; Yang et al., 2023b) (Fig. 1).

Ubiquinone, commonly known as coenzyme Q or CoQ, is a lipophilic metabolite characterized by its unique structure comprising a redox-active quinone head group and an extensive polyisoprenoid lipid tail (Guerra and Pagliarini, 2023). Beyond its well-known role in mitochondrial electron transport, when existing in its reduced form as CoQH_2_, this remarkable molecule can also act as an RTA for cellular defense against ferroptosis (Guerra and Pagliarini, 2023). FSP1 operates as a nicotinamide adenine dinucleotide (NADH)/nicotinamide adenine dinucleotide phosphate (NADPH)-dependent oxidoreductase that utilizes the reducing power of NADH and/or NADPH to convert CoQ to its protective reduced form, CoQH_2_, thereby effectively suppressing ferroptosis independently of GPX4. Conversely, FSP1 deletion or inhibition sensitizes cancer cells to ferroptosis (Bersuker et al., 2019; Doll et al., 2019; Nakamura et al., 2023). FSP1 is thought to reduce the nonmitochondrial pool of CoQ to CoQH_2_, exerting its antiferroptosis role mainly on the plasma membrane (Bersuker et al., 2019; Doll et al., 2019). In contrast, CoQ synthesis and its subsequent reduction to CoQH_2_ predominantly take place within the mitochondria, with the conversion of CoQ to CoQH_2_ orchestrated by a series of enzymes located on the inner mitochondrial membrane, including DHODH and GPD2 (Guerra and Pagliarini, 2023). Correspondingly, the inactivation or deletion of DHODH or GPD2 leads to heightened ferroptosis sensitivity in cancer cells due to the decreased production of CoQH_2_ within the mitochondria (Mao et al., 2021; Wu et al., 2022). The subcellular segregation of FSP1 from DHODH, GPD2, and CoQ synthesis underscores the potential role of CoQ compartmentalization or transport in the regulation of ferroptosis (Gan, 2021). In support of this hypothesis, another study determined that StAR-related lipid transfer domain-containing 7 (STARD7), a lipid transfer protein, serves as a mediator for the transport of CoQ from the mitochondria to the plasma membrane, facilitating its utilization by FSP1 for ferroptosis defense on the plasma membrane (Deshwal et al., 2023).

It is noteworthy that the role of DHODH in ferroptosis has been debated (Mao et al., 2023; Mishima et al., 2023), although other recent studies have not only solidified its significance in ferroptosis defense but have also provided new mechanistic insights (Yang et al., 2023a; Zhan et al., 2023). One study discovered that despite their localization in different subcellular compartments, mitochondrial DHODH and cytosolic enzymes, including carbamoyl-phosphate synthetase II, aspartate transcarbamoylase, and dihydroorotase (CAD) and uridine 5ʹ monophosphate synthase (UMPS) can form a multienzyme complex through the bridging protein voltage-dependent anion-selective channel protein 3 (VDAC3), positioned across the outer mitochondrial membrane. The formation of this complex, termed the pyrimidinosome, facilitates substrate channeling and promotes efficient pyrimidine biosynthesis and ferroptosis defense. Consequently, a genetic deficiency of DHODH or its upstream proteins, such as CAD or VDAC3, leads to an increased sensitivity to ferroptosis (Yang et al., 2023a). Another study showed that lysyl oxidase-like 3 (LOXL3) inhibits ferroptosis and fosters chemoresistance in cancer cells by stabilizing DHODH, unveiling a regulatory mechanism in the DHODH-mediated ferroptosis defense (Zhan et al., 2023).

BH_4_, recognized as a cofactor for aromatic amino acid hydroxylases and various enzymes, has also emerged as a potent RTA with notable ferroptosis-suppressing capabilities (Soula et al., 2020). The depletion of GCH1, the metabolic enzyme responsible for the rate-limiting step in the BH_4_ biosynthesis pathway, renders cancer cells more vulnerable to ferroptosis (Kraft et al., 2020; Soula et al., 2020). It is worth noting that other functions of GCH1, such as the regulation of the production of CoQH_2_ and PLs containing two PUFA tails, might also contribute to its ferroptosis-suppressing role (Kraft et al., 2020).

Additional research identified the reduced forms of vitamin K as robust RTAs in the defense against ferroptosis and found that FSP1 plays a pivotal role in mediating vitamin K reduction (Mishima et al., 2022). Therefore, FSP1’s dual roles in reducing both CoQ and vitamin K contribute to its potent antiferroptosis function. It is important to note that, in the canonical vitamin K cycle, the reduction of vitamin K is primarily mediated by other metabolic enzymes such as VKORC1L1. Consistent with this, VKORC1L1 has been shown to suppress ferroptosis by producing the reduced forms of vitamin K independently of GPX4 or FSP1 (Yang et al., 2023b).

Collectively, these recent studies have shed significant light on the pivotal role of RTAs in the defense against ferroptosis and have provided novel insights into the intricate mechanisms that counteract lipid peroxidation. Future research endeavors will aim to identify additional RTAs, elucidate the functions of the enzymes involved in producing such RTAs, and explore the intricate coordination among these factors in ferroptosis defense.

### Ferroptosis in cancer therapy

Ferroptosis, akin to apoptosis, serves as a pivotal mechanism in tumor suppression, involving a range of tumor suppressor and oncogenic proteins that promote and suppress ferroptosis, respectively. A prominent example is p53, the most commonly mutated tumor suppressor, known to hinder tumor growth, at least in part, by inducing ferroptosis (Liu and Gu, 2022). This is achieved through the transcriptional modulation of various ferroptosis regulatory proteins, such as SLC7A11, calcium-independent phospholipase A2β (iPLA2β), and VKORC1L1 (Chen et al., 2021a; Jiang et al., 2015; Liu and Gu, 2022; Yang et al., 2023b). (Of note, additional studies have revealed a ferroptosis suppressive function of p53 under certain conditions (Tarangelo et al., 2018; Xie et al., 2017), suggesting a context-dependent role of p53 in ferroptosis regulation (Liu and Gu, 2022) Other tumor suppressors, such as BAP1, fumarase, and kelch-like ECH-associated protein 1 (KEAP1), have similarly been demonstrated to promote ferroptosis in cancer cells (Gao et al., 2019; Koppula et al., 2022; Sun et al., 2016; Zhang et al., 2018). Conversely, the p53 R175H mutant, which has oncogenic gain-of-function properties, suppresses ferroptosis by abolishing BTB and CNC homology 1 (BACH1)-mediated downregulation of SLC7A11 expression, leading to enhanced tumor growth (Su et al., 2023). Furthermore, the oncogenic activation of phosphoinositide 3-kinase (PI3K)- mTORC1 signaling has been shown to promote ferroptosis resistance in cancer cells (Yi et al., 2020).

While the loss of tumor suppressors or the activation of oncogenes frequently results in ferroptosis resistance in cancer, certain genetic or metabolic alterations can enhance the sensitivity of cancer cells to ferroptosis (Lei et al., 2022). For example, in the E-cadherin-neurofibromin 2 (NF2-Hippo pathway, the deficiency of tumor suppressors such as NF2 renders cancer cells or tumors susceptible to ferroptosis (Wu et al., 2019; Yang et al., 2019). This vulnerability may offer a promising therapeutic avenue through which ferroptosis-inducing drugs can be strategically employed to target tumors with mutations in this pathway, and preclinical studies in *NF2*-mutant mesotheliomas support this notion (Wu et al., 2019). Another illustrative example comes from studies of neuroblastomas, in which *MYCN* amplification plays a critical role in disease progression. Recent investigations have demonstrated that MYCN-high cancer cells exhibit heightened ferroptosis sensitivity compared with their MYCN-low counterparts, paving the way for the development of a targeted ferroptosis-inducing therapy for effectively treating MYCN-high neuroblastomas (Alborzinia et al., 2022). Lastly, certain therapy-resistant cancer cells, exemplified by therapy-resistant mesenchymal-type cells and drug-tolerant persister cells, frequently undergo distinct metabolic adaptations. For instance, increased levels of PUFA-PLs, which are often associated with epithelial-to-mesenchymal transition, become a defining feature in these cells (Hangauer et al., 2017; Viswanathan et al., 2017). Such altered metabolic states create a dependency on the GPX4-mediated defense against ferroptosis for their survival. As a result, these cells become particularly susceptible to ferroptosis triggered by GPX4 inhibition, presenting a potential vulnerability that could be harnessed for therapeutic interventions (Hangauer et al., 2017; Viswanathan et al., 2017).

The identification of these vulnerabilities to ferroptosis across various cancer contexts has ignited significant interest in the development and evaluation of FINs for potential applications in cancer therapy. A range of compounds exhibiting ferroptosis-inducing capabilities have been identified. Many of these FINs specifically target proteins involved in GPX4-dependent ferroptosis defense mechanisms (Feng and Stockwell, 2018). For instance, erastin, imidazole ketone erastin (IKE), sulfasalazine, and sorafenib—which inhibit SLC7A11—and cyst(e)inase—which breaks down extracellular cystine and cysteine—are collectively categorized as class I FINs (Cramer et al., 2017; Feng and Stockwell, 2018). However, the categorization of sorafenib as a FIN has been challenged by another study (Zheng et al., 2021). The ferroptotic effects of sorafenib might be cell line- or context-dependent. Similarly, GPX4 inhibitors, including RSL3, ML162, ML210, and JKE-1674, constitute class II FINs (Feng and Stockwell, 2018). The array of FINs is further expanded by compounds such as FIN56, a class II FIN that depletes both GPX4 protein and CoQ, as well as class IV FINs such as FINO_2_, which oxidize iron and indirectly inhibit GPX4 (Feng and Stockwell, 2018).

It is important to note that most of these FINs are applicable only for cell line studies; they lack the solubility and favorable pharmacokinetics needed for effective animal treatment (Feng and Stockwell, 2018). This limitation restrains their further clinical application. In current preclinical investigations, the focus has mainly revolved around several class I FINs, namely IKE, sulfasalazine, sorafenib, and cyst(e)inase. The outcomes of these animal studies have demonstrated that, apart from in specific tumor contexts that display heightened vulnerability to ferroptosis, these FINs generally yield only moderate antitumor effects when employed as standalone treatments (Chen et al., 2021d; Lei et al., 2022). This observation has catalyzed the exploration of synergistic approaches involving the combination of FINs with established standard-of-care therapies including radiotherapy, immunotherapy, targeted therapy, and chemotherapy (Lei et al., 2022). Intriguingly, some of these conventional therapies themselves, such as radiotherapy and immunotherapy, are capable of inducing lipid peroxidation and ferroptosis (Lang et al., 2019; Lei et al., 2020, 2021; Wang et al., 2019; Ye et al., 2020). Hence, the integration of FINs with these therapeutic modalities often produces a synergistic effect that bolsters the induction of ferroptosis within tumors and thereby enhances the overall therapeutic efficacy (Lei et al., 2022).

The dearth of effective GPX4 inhibitors suitable for *in vivo* treatment has long stood as a significant hurdle in the field of ferroptosis translational research. However, a recent breakthrough in this regard comes in the form of JKE-1674, a newly developed GPX4 inhibitor that exhibits improved pharmacokinetic properties (Eaton et al., 2020). Encouragingly, this inhibitor has demonstrated some therapeutic effects within *RB1*-deficient prostate tumors, igniting hope for its potential clinical applicability (Wang et al., 2023a). The continued advancement and refinement of GPX4 inhibitors will enrich the repertoire of tools available for exploring ferroptosis induction as a therapeutic strategy in cancer treatment.

Furthermore, another important avenue for future exploration lies in understanding the implications of ferroptosis-inducing therapy within the intricate landscape of the tumor microenvironment, particularly the immune system (Lei et al., 2022; Tang et al., 2023; Xu et al., 2021). The role of ferroptosis in the immune system is complex, and a detailed discussion of it extends beyond the scope of this review. We refer readers to several comprehensive reviews on this specific topic (Lei et al., 2022; Tang et al., 2023; Xu et al., 2021). In brief, the interplay between ferroptotic cancer cells and immune cells is bidirectional: ferroptotic cancer cells can demonstrate either immune-suppressive or immune-promoting effects, while immune cells are capable of either promoting or inhibiting ferroptosis in cancer cells. These dynamics are context-dependent and hinge upon the specific functionalities of the immune cells involved. Adding another layer of complexity, different immune cell types exhibit varying sensitivities to ferroptosis. Consequently, whereas FINs can target tumor cells effectively, their potential impact on the survival and functionality of immune cells must be taken into consideration (Xu et al., 2021). The prospect of modulating immune cell viability through the actions of FINs raises the concern of potentially compromising the overall therapeutic efficacy of ferroptosis-inducing treatments (Lei et al., 2022; Xu et al., 2021). This multifaceted consideration underscores the complexity of designing strategies that balance tumor cell destruction with immune cell preservation.

Adding to this complexity, a contentious issue has arisen from discrepant findings pertaining to the impact of FINs or ferroptosis inhibitors on tumor growth in preclinical models with intact immune systems. Although several studies have substantiated the antitumor effects of FINs, alongside the tumor-promoting effects of ferroptosis inhibitors (Badgley et al., 2020; Lang et al., 2019; Liao et al., 2022; Wang et al., 2019, 2023a), a contrasting study has revealed an unexpected antitumor effect achieved through ferroptosis inhibition (Kim et al., 2022). This dichotomy of outcomes necessitates an in-depth exploration to unravel the intricate interplay among ferroptosis modulation, immune responses, and tumor development within the tumor microenvironment. Finally, future studies utilizing preclinical models that faithfully mimic the intricate interactions within the tumor microenvironment will be critical for ferroptosis translational research.

## Cuproptosis

Copper, an indispensable trace nutrient, plays a critical role as a catalytic cofactor across an array of biological processes, such as antioxidant defense, mitochondrial respiration, and the synthesis of vital biomolecules (Cobine et al., 2021). Nevertheless, the dual nature of copper emerges when its concentration surpasses the threshold maintained by intricate homeostatic mechanisms that have evolved to avert toxicity (Royer and Sharman, 2023). Recent studies have revealed a novel facet of copper biology: excessive cellular copper levels can trigger a distinct form of regulated cell death, termed “cuproptosis” (Tsvetkov et al., 2022). Given that cuproptosis has a profound connection with mitochondrial metabolism and has been particularly observed in cancer cells reliant on aerobic respiration, targeting cuproptosis has emerged as a tantalizing avenue for potential anticancer therapeutic interventions. In the following section, we discuss the intricate landscape of copper metabolism regulation, delve into the current understanding of cuproptosis, and explore its potential in cancer therapy.

### Copper metabolism regulation

Similar to the levels of intracellular iron, the levels of intracellular copper undergo stringent regulation through a complex interplay of processes encompassing copper uptake, utilization, storage, and export. To lay the foundation for our subsequent discussion on cuproptosis, here, we offer a concise overview of the regulatory mechanisms governing copper metabolism. For a more detailed understanding of copper metabolism, we refer readers to recent comprehensive reviews on this subject (Chen et al., 2022; Ge et al., 2022).

**Figure 2.**
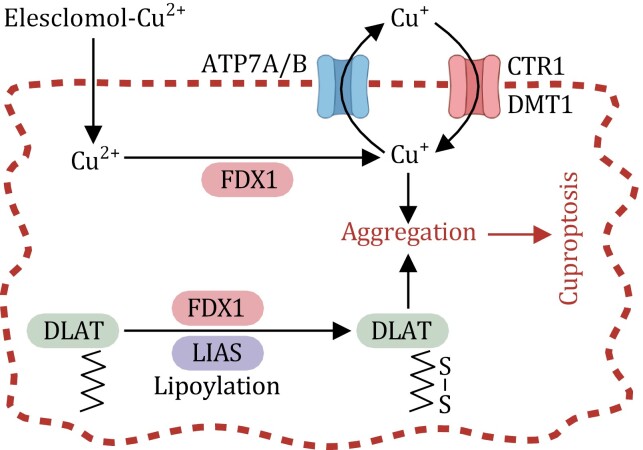
**Key regulators of cuproptosis.** Extracellular copper is transported to the inside of the cell through copper ionophores (e.g., elesclomol) or transporters (CTR1 and DMT1). ATP7A/B are responsible for the efflux of copper. FDX1 reduces Cu^2+^ to Cu^+^, and interacts with LIAS to mediate protein lipoylation of a few key metabolic enzymes such as DLAT. It is proposed that a direct binding between Cu^+^ and lipoylated proteins induces the oligomerization of the lipoylated proteins, which in turn triggers a toxic gain-of-function effect and ultimately induces cuproptosis.

Copper uptake into cells is primarily facilitated by copper transport 1 (CTR1), also known as solute carrier family 31 member 1 (SLC31A1) (Kuo et al., 2001) (Fig. 2). The cellular dynamics of copper uptake are governed by sophisticated adaptive mechanisms that involve the modulation of CTR1 expression levels in response to intracellular copper concentrations: the expression of CTR1 is upregulated in response to copper depletion or downregulated when confronted with copper excess (Kuo et al., 2012). Although CTR1 serves as a principal conduit for cellular copper entry, divalent metal transporter 1 (DMT1, also known as solute carrier family 11 member 2 or SLC11A2; Fig. 2) can contribute to copper uptake under specific circumstances, particularly in situations in which CTR1 is lacking. As a result, the simultaneous depletion of both CTR1 and DMT1 is necessary to completely suppress copper uptake in certain cell types (Lin et al., 2015).

Once copper enters the intracellular milieu, it is transported to specific subcellular destinations, where it will be either harnessed for vital cellular functions or managed to prevent toxicity. This intricate process is orchestrated by a spectrum of chaperone proteins. Cytochrome c oxidase copper chaperone (COX17), for instance, ferries copper to cytochrome c oxidase (CCO), which acts as an indispensable constituent of the mitochondrial electron transport chain and relies on copper to carry out its essential function in oxidative phosphorylation (Palumaa et al., 2004). Another noteworthy player in copper distribution is the copper chaperone for superoxide dismutase (CCS). By shuttling copper to superoxide dismutase 1 (SOD1), an antioxidant enzyme important in thwarting oxidative stress, CCS contributes to the cellular defense against reactive oxygen species (Wright, 2020). Furthermore, the antioxidant 1 copper chaperone (ATOX1) transports copper to ATPase copper transporting α and β (ATP7A and ATP7B), transmembrane proteins specializing in copper export (see below), thereby ensuring the controlled elimination of excess intracellular copper (Hatori and Lutsenko, 2016).

It is critical to effectively manage any surplus copper that might accumulate within cells and induce potential toxicity. In this regard, copper storage proteins bind and sequester excess copper ions and thereby avert potential harm. The metallothionein (MT) family proteins stand as a prime example of such proteins (Tapia et al., 2004).

Excess intracellular copper can also be mitigated through copper export. ATP7A and ATP7B are integral transmembrane proteins that serve as key players in copper export by critically contributing to the maintenance of copper equilibrium within cells (Schmidt et al., 2018) (Fig. 2). These proteins predominantly localize to the trans Golgi network (TGN), where they actively transport copper from the cytosol into the luminal space of the TGN. This orchestrated transport not only sustains copper homeostasis but also furnishes copper to copper-dependent enzymes situated within the secretory pathway. An increase in intracellular copper concentrations triggers a dynamic repositioning of ATP7A and ATP7B, causing these proteins to translocate from the TGN to vesicular compartments that subsequently merge with the plasma membrane. This process facilitates the export of copper from cells. Upon the restoration of copper concentrations to a physiological state, these proteins relocalize back to the TGN (Hasan et al., 2012). Mutations in *ATP7A* and *ATP7B* lead to disturbances in copper homeostasis, resulting Menkes disease and Wilson disease, respectively (de Bie et al., 2007).

The proteins engaged in copper metabolism act in concert to maintain the delicate balance of intracellular copper levels. Nevertheless, a disruption of the homeostasis of copper metabolism can lead to the excessive accumulation of copper within cells, ultimately contributing to cuproptotic cell death, as discussed below.

### Cuproptosis linking copper toxicity to mitochondrial lipoylation

The approaches to inducing copper-triggered cell death and ferroptosis are notably different. Ferroptosis is primarily induced through genetic or pharmacological interventions that disturb cellular defense mechanisms against lipid peroxidation, rather than by inundating cells with iron. In contrast, copper-induced cell death involves directly overwhelming cells with excessive copper, such as by treating cells with copper and copper ionophores (small molecules that facilitate the entry of copper into cells), which has provoked robust cell death in multiple cancer cell lines. Copper-induced cell death was found to be resistant to the known cell death inhibitors and did not display the characteristics of other well-known cell death modes, such as caspase 3 cleavage (Tsvetkov et al., 2022). Therefore, the term “cuproptosis” was coined to describe this distinct form of cell death (Tsvetkov et al., 2022).

Notably, compared with their glycolytic counterparts, cancer cells reliant on mitochondrial respiration exhibit a heightened sensitivity to cuproptosis. Furthermore, it has been shown that the use of inhibitors targeting mitochondrial electron transport chain complexes suppresses cuproptosis; this finding established the intimate connection between cuproptosis and mitochondrial metabolism (Tsvetkov et al., 2022). In-depth investigations utilizing CRISPR screens identified the key regulators of cuproptosis. Among these regulators, ferredoxin 1 (FDX1) emerged as a central player that is required for cuproptosis (Tsvetkov et al., 2022). Interestingly, FDX1 was previously identified as a target of the copper ionophore elesclomol and was also shown to reduce Cu^2+^ to its more toxic form Cu^1+^; subsequent investigations have unveiled its hitherto-undiscovered function in governing protein lipoylation (Tsvetkov et al., 2019, 2022) (Fig. 2). This is achieved through its interaction with lipoic acid synthetase (LIAS), an enzyme that catalyzes the final step in lipoic acid biosynthesis and acts as a pivotal upstream regulator of protein lipoylation (Dreishpoon et al., 2023) (Fig. 2). Protein lipoylation is a posttranslational modification entailing the covalent linkage of lipoamide to a specific lysine residue in target proteins. This modification is integral to the functionality of four key metabolic enzymes that regulate the mitochondrial tricarboxylic acid (TCA) cycle, including dihydrolipoamide S-acetyltransferase (DLAT), an important subunit of the pyruvate dehydrogenase complex (Rowland et al., 2018) (Fig. 2). The genetic depletion of LIAS and DLAT, like FDX1 deletion, has been found to render cancer cells resistant to cuproptosis (Tsvetkov et al., 2022). Interestingly, despite their indispensability for cuproptosis, the levels of FDX1, LIAS, and protein lipoylation were notably diminished during the process (Tsvetkov et al., 2022). This phenomenon might reflect an adaptive but ultimately futile cellular attempt to counteract the toxic effects of excessive copper by dampening protein lipoylation. These findings collectively underscore the critical role of protein lipoylation in cuproptosis.

The precise mechanisms by which the cellular processes downstream of protein lipoylation instigate cuproptosis remain unclear. Notably, a direct binding between copper and lipoylated DLAT has been observed, prompting the oligomerization of the lipoylated protein during cuproptosis (Tsvetkov et al., 2022). This phenomenon raises the intriguing possibility that the resulting protein aggregates could elicit a toxic gain-of-function effect, ultimately culminating in the execution of cuproptotic cell death (Fig. 2). These intriguing findings present a compelling avenue for future exploration into this unique and complex mode of cellular demise. Finally, the potential role of the proteins involved in copper metabolism (as discussed in the preceding section) in regulating cupropotosis remains to be studied.

### Copper ionophores and cuproptosis in cancer therapy

In recent years, a growing body of research has underscored the promise of targeting copper metabolism as a strategy in anticancer therapeutics (Ge et al., 2022). The notion of using copper overload to facilitate the accumulation of free radicals within cancer cells has drawn attention due to its potential in driving copper-induced cell death. The emergence of cuproptosis as a distinct cell death mechanism has further expanded the horizons for employing copper ionophores as inducers of this cell death modality in cancer treatment. This development parallels the approach of utilizing ferroptosis inducers in cancer therapy, thereby expanding innovative therapeutic avenues.

A notable exemplar in this context is the copper ionophore elesclomol, which has demonstrated the ability to selectively ferry copper to mitochondria, triggering cell death, now recognized as cuproptosis (Nagai et al., 2012; Tsvetkov et al., 2019, 2022; Zheng et al., 2022). Interestingly, the transition of cancer cells from glycolysis to oxidative phosphorylation renders them more resistant to proteasome inhibitors, yet uniquely susceptible to the actions of elesclomol (Tsvetkov et al., 2019, 2022).

In an initial phase I clinical trial, elesclomol demonstrated a favorable safety profile but yielded no clinical response when administered as a single agent to patients with refractory acute myeloid leukemia (Hedley et al., 2016). A phase II clinical trial focusing on patients with stage IV melanoma showed that elesclomol enhanced the therapeutic efficacy of paclitaxel and had an acceptable toxicity profile (O’Day et al., 2009). However, a following phase III trial investigating the use of elesclomol in patients with melanoma showed that the combination of elesclomol and paclitaxel did not extend progression-free survival among patients with advanced melanoma; a subsequent analysis revealed that elesclomol exhibited more robust antitumor activity in patients with lower plasma lactate dehydrogenase (LDH) levels, a possible indicator of lower glycolysis rates in these individuals (O’Day et al., 2013). Notably, this clinical observation aligns with studies conducted in cancer cell lines, which discovered that elesclomol induces more potent cuproptosis in cancer cells reliant on mitochondrial respiration—in other words, those cancer cells that are less glycolytic—compared with their glycolytic counterparts (Tsvetkov et al., 2019, 2022). These findings, therefore, suggest the potential application of LDH levels and/or other cuproptosis markers as biomarkers for patient selection in elesclomol therapy.

Another illustration involves disulfiram, a medication that inhibits aldehyde dehydrogenase (ALDH) activity (Veverka et al., 1997). Other studies have uncovered an additional facet of disulfiram’s action: it operates as a copper-binding compound, releasing copper within a reducing intracellular environment, thus augmenting copper levels within cells (Li et al., 2020). Recent investigations have demonstrated that the combined application of disulfiram and copper incites cuproptosis and manifests antitumor effects that are selectively directed at ALDH^+^ cancer stem cells. This therapeutic strategy holds the promise of mitigating the likelihood of tumor recurrence (Gan et al., 2023; Li et al., 2020). Moreover, the disulfiram-copper complex has exhibited the ability to effectively counteract drug resistance in doxorubicin-resistant oral cancer cells, leading to cell death (Chen et al., 2021b).

In clinical studies, disulfiram’s potential has been reinforced by its ability to traverse the blood–brain barrier. Its anticancer and chemo-sensitizing effects have been confirmed in patients with glioblastoma (Triscott et al., 2012). Additionally, a phase I clinical trial underscored the potency of the disulfiram-copper complex when combined with temozolomide, revealing an enhancement in the progression-free survival rates among glioblastoma patients treated with this therapy (Huang et al., 2016).

Lastly, NSC319726 has emerged as another intriguing copper ionophore for cancer therapy. Originally identified as a reactivator of the p53^R175H^ mutant protein, NSC319726 was found to exert cytotoxic effects specifically against tumors harboring the *p53*^*R172H*^ mutation (Yu et al., 2012). Recent investigations have demonstrated an additional facet of NSC319726’s capabilities: it functions as a copper ionophore, capable of inducing robust cell death in cancer cells through copper binding (Shimada et al., 2018; Tsvetkov et al., 2022). Furthermore, NSC319726’s interaction with copper stimulates the generation of reactive oxygen species and leads to the depletion of deoxyribosyl purines. These actions collectively contribute to the induction of cell cycle arrest in tumor cells derived from glioblastoma patients (Shimada et al., 2018). These studies highlight the potential of NSC319726 as a promising candidate for anticancer therapies.

Collectively, these investigations underscore the considerable potential held by a variety of copper ionophores as promising cancer therapeutics. However, it is noteworthy that most of these studies were conducted prior to the recognition of cuproptosis, and these compounds often exhibit multifaceted mechanisms of action. Therefore, the extent to which the antitumor effects of these compounds are attributed to their capacity to induce cuproptosis remains a subject of inquiry. Future explorations will be directed toward elucidating the role of cuproptosis in orchestrating the antitumor efficacy of these copper ionophores, as well as revealing additional strategies that leverage cuproptosis for cancer treatment.

## Disulfidptosis

### Disulfidptotic cell death resulting from excessive disulfide stress

As discussed in the “Ferroptosis” section, SLC7A11 is a pivotal player in ferroptosis suppression and acts by facilitating the import of cystine to drive GSH synthesis and bolstering GPX4-mediated defense mechanisms against ferroptosis. However, SLC7A11 overexpression has been shown to paradoxically induce robust cell death under conditions of glucose deprivation (Goji et al., 2017; Joly et al., 2020; Koppula et al., 2017; Liu et al., 2020b, 2023a; Shin et al., 2017). This form of cell demise has recently been recognized as a regulated cell death modality distinct from others such as apoptosis and ferroptosis, and the term “disulfidptosis” was subsequently coined to underscore the underlying concept that this type of cell death results from excessive disulfide stress (Liu et al., 2023a). The dual role of SLC7A11 in governing both ferroptosis and disulfidptosis is somewhat analogous to the established opposing functions of caspase-8 in the regulation of apoptosis and necroptosis, wherein caspase-8 activation prompts apoptosis while concurrently dampening receptor-interacting protein kinase 1 (RIPK1)-mediated necroptosis (Tummers and Green, 2017). This analogy reinforces the intricate interplays and complexities inherent in the regulation of various modes of cell death. Below, we discuss the basic concept and known mechanisms of disulfidptosis. For a more comprehensive exploration of this cell death mechanism, we direct readers to a recent review (Liu et al., 2023b).

From a metabolic perspective, disulfidptosis underscores the metabolic trade-off that cancer cells with high expression of SLC7A11 (SLC7A11-high cells), have to contend with due to their elevated cystine uptake (Liu et al., 2021). Cystine, an amino acid characterized by its extremely low solubility, can pose significant toxicity to cells when it accumulates to high levels within the cytosol (Liu et al., 2020b). SLC7A11-high cells transport substantial quantities of cystine into their cytosol, necessitating a rapid conversion of cytosolic cystine into the much more soluble cysteine, and this conversion requires NADPH as a critical reducing agent. (Subsequently, intracellular cysteine acts as a key precursor for the synthesis of GSH.) However, this reduction process also comes at a cost, as it causes the high consumption of the cytosolic pool of NADPH (Goji et al., 2017; Joly et al., 2020; Liu et al., 2020b), which is primarily generated from glucose through the pentose phosphate pathway (Chen et al., 2019).

**Figure 3.**
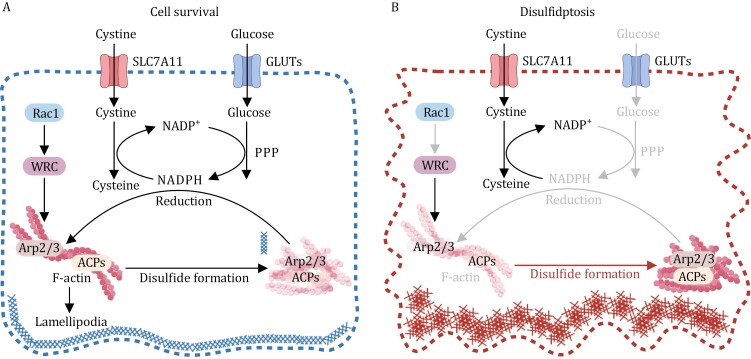
**Disulfidptosis regulation mechanism.** (A) Cells with high SLC7A11 expression (SLC7A11-high cells) experience high rates of cystine uptake and subsequent reduction to cysteine. The latter reaction requires NADPH as a reducing agent, which is mainly derived from glucose via the PPP. The presence of ample glucose is required for preventing aberrant accumulation of intracellular cystine and the formation of disulfide bonds in actin cytoskeleton proteins and maintaining cell survival in SLC7A11-high cells. (B) Under conditions of glucose deprivation, where NADPH supply is limited, SLC7A11-high cells experience an excessive accumulation of cystine and other disulfide molecules. As a result, abnormal disulfide bond formation occurs within actin cytoskeleton proteins, ultimately leading to the collapse of the actin network and disulfidptosis. It is likely that Rac1-WRC-Arp2/3-mediated branched actin polymerization and lamellipodia formation create a favorable environment for the formation of disulfide bonds among actin cytoskeleton proteins, thereby facilitating the process of disulfidptosis.

Consequently, SLC7A11-high cancer cells exhibiting substantial cystine uptake and reduction become highly reliant on glucose to provide the necessary NADPH and maintain the swift conversion of cystine to cysteine (Fig. 3A). When deprived of glucose and, consequently, an NADPH supply, these cells encounter NADPH depletion. This leads to an abnormal buildup of intracellular cystine and other disulfide molecules, culminating in a state of disulfide stress. Ultimately, this disulfide stress triggers rapid disulfidptotic cell death (Goji et al., 2017; Joly et al., 2020; Liu et al., 2020b, 2021, 2023a) (Fig. 3B).

The intricate mechanisms through which disulfide stress gives rise to cell death are still not fully understood. Recent investigations, however, have proposed a conceptual framework for the underlying process (Liu et al., 2023a). In glucose-deprived SLC7A11-high cells, disulfide stress has been shown to trigger aberrant disulfide bonding in actin cytoskeleton proteins. This, in turn, disrupts the actin network’s structure and its detachment from the plasma membrane, ultimately culminating in disulfidptotic cell death (Liu et al., 2023a). A significant contributing factor in this mechanism appears to be Rac-WAVE regulatory complex (WRC)-mediated branched actin polymerization and lamellipodia formation. This process has been shown to enhance disulfidptosis, possibly due to the susceptibility of the branched actin network in lamellipodia to disulfide bonding among actin cytoskeleton proteins (Fig. 3B). Consistent with this model, the genetic depletion of components within the WRC complex suppressed disulfidptosis, whereas the constitutive activation of Rac promoted it (Liu et al., 2023a).

Additional research has revealed that the occurrence of disulfide stress and subsequent disulfidptosis is not limited solely to glucose deprivation. Studies have demonstrated that SLC7A11-high cells can also undergo disulfidptosis under conditions involving hydrogen peroxide (H_2_O_2_) treatment or the inhibition of thioredoxin reductase 1 (TXNRD1), an enzyme responsible for catalyzing the reduction of cystine to cysteine (Yan et al., 2023; Zhong et al., 2023). These findings broaden the range of contexts in which disulfidptosis can manifest. It is worth noting that, although both glucose starvation and H_2_O_2_ treatment can trigger cell death in SLC7A11-deficient or SLC7A11-low cells, the onset of cell death is notably delayed, and the mechanisms underlying these delayed cell death responses involve different modes such as apoptosis and/or necroptosis; high expression of SLC7A11 switches cell death to more rapid disulfidptosis under these metabolic stress conditions (Jantas et al., 2020; Liu et al., 2023a; Yan et al., 2023). This underscores the intricate interplay between SLC7A11 expression levels, the cellular redox balance, and the spectrum of cell death pathways, adding another layer of complexity to the regulatory mechanisms governing cell fates.

### Disulfidptosis in cancer therapy

These recent investigations have collectively unveiled disulfidptosis as a distinct form of regulated cell death that arises from the dynamic interplays among cystine uptake, NADPH availability, and the management of disulfide stress. This revelation further highlights a metabolic vulnerability of SLC7A11-high cancer cells, illuminating potential therapeutic avenues by targeting their dependence on glucose and NADPH for survival. As elucidated earlier, SLC7A11’s role in cystine uptake provides a powerful protective effect against ferroptosis, and SLC7A11 has also been shown to suppress apoptosis (Koppula et al., 2021b). As a result, SLC7A11-high tumors likely exhibit resistance against therapies that are designed to induce ferroptosis and/or apoptosis. However, the emerging susceptibility of these cancer cells to disulfidptosis introduces a new avenue for therapeutic intervention in SLC7A11-high malignancies.

Indeed, preclinical investigations have demonstrated that, compared to their SLC7A11-low counterparts, SLC7A11-high cancer cells and tumors manifest heightened sensitivity to inhibitors of glucose transporters (GLUTs) (Joly et al., 2020; Liu et al., 2020b, 2023a). Notably, the inhibition of glucose transporters leads to robust cell death in SLC7A11-high cancer cells, and this process is potentially mediated by the disulfidptosis mechanism (Liu et al., 2023a). These findings indicate that targeting disulfidptosis could hold significant promise in the treatment of cancers that display this particular metabolic vulnerability, such as SLC7A11-high tumors. An illustrative example of such cancer types is *KEAP1*-mutant lung cancer, in which *KEAP1* loss-of-function mutation leads to stabilized NRF2, which in turn enhances the transcription of *SLC7A11* and results in its high expression (Rojo de la Vega et al., 2018). Intriguingly, *KEAP1*-mutant or *KEAP1*-deficient lung cancer cells exhibit an increased reliance on glucose, and under glucose-deprived conditions, they accumulate disulfide molecules aberrantly due to upregulated SLC7A11-mediated cystine uptake (Koppula et al., 2021a). This vulnerability makes *KEAP1*-mutant lung cancer cells or tumors sensitive to glucose transporter inhibition and suggests a potential therapeutic strategy to target disulfidptosis in this subset of lung cancers (Koppula et al., 2021a). Given the current absence of a disulfidptosis inhibitor or biomarker for *in vivo* analyses, further investigations are needed to determine whether the heightened sensitivity to glucose transporter inhibition observed in *KEAP1*-mutant or SLC7A11-high tumors involves additional mechanisms. Further exploration and validation of disulfidptosis-targeting strategies in preclinical and clinical settings could usher in a new era of precision medicine for SLC7A11-high cancers.

## Lysozincrosis

During the course of cancer development, there is often an upregulation of lysosomal function to fulfill the increased energy demands of rapidly proliferating cancer cells (Tang et al., 2020). An important player in this context is the transient receptor potential mucolipin 1 (TRPML1), a cation channel with dual permeability to Ca^2+^ and Zn^2+^. TRPML1 predominantly localizes on intracellular vesicular membranes, particularly lysosomes (Devinney et al., 2009), and is involved in many key lysosomal functions, including membrane trafficking, exocytosis, lysosomal biogenesis, and heavy-metal homeostasis (Gurunathan et al., 2021). Notably, the upregulation of TRPML1 expression is pronounced in metastatic melanoma cells and has been linked to conferring growth advantages to these aggressive cancer cells (Du et al., 2021).

Zn^2+^, an indispensable trace element essential for normal cellular processes, is intricately tied to these mechanisms (Yamaguchi et al., 2009). However, the excessive release of intracellular Zn^2+^ can prove toxic, hampering mitochondrial function and leading to mitochondrial dysfunction, cellular energy depletion, and eventual cell death (Chimienti et al., 2001).

**Figure 4.**
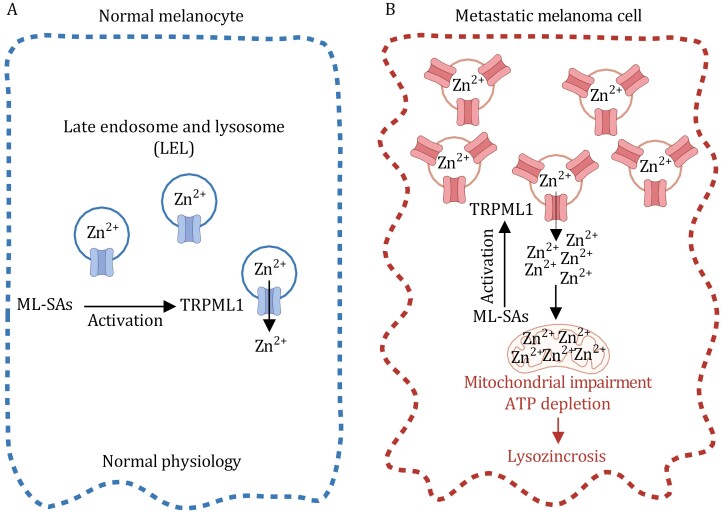
**Key regulators of lysozincrosis.** (A) Normal melanocytes exhibit relatively moderate expression of TRPML1 and therefore no significant vulnerability to ML-SA treatment. (B) In metastatic melanoma cells (which exhibit high expression levels of TRPML1), ML-SA administration results in TRPML1 hyperactivation and lysosomal Zn^2+^ release, leading to mitochondrial impairment, ATP depletion, and lysozincrosis.

Recent investigations have revealed the intriguing potential of TRPML-specific synthetic agonists (ML-SAs) as inducers of rapid lysosomal Zn^2+^-dependent cell death in metastatic melanoma cells but not in normal cells (Du et al., 2021) (Fig. 4A and 4B). This unique form of cell death has been coined “lysozincrosis.” At a mechanistic level, the activation of TRPML1 by ML-SAs prompts the release of Zn^2+^ from lysosomes, culminating in mitochondrial impairment and the swift depletion of ATP and ultimately leading to lysozincrosis (Fig. 4B). Importantly, cells with high TRPML1 expression exhibit a distinct susceptibility to lysozincrosis. In a murine melanoma model, ML-SAs exhibited significant potency in curbing tumor progression *in vivo*, offering a selective eradication of metastatic melanoma cells while leaving normal cells unharmed (Du et al., 2021). Therefore, the pharmacological harnessing of TRPML1 through the activation of lysozincrosis is a promising avenue for potential therapeutic strategies, particularly in the context of metastatic melanoma and potentially for other malignancies.

## Alkaliptosis

The disruption of the intracellular pH balance can lead to either acidification or alkalization, culminating in cell death. While intracellular acidification has been associated with apoptosis, necroptosis, and autophagic cell death (Lagadic-Gossmann et al., 2004; Wang et al., 2015; Xu et al., 2011), the role of intracellular alkalization in cell death remains relatively unexplored. An intriguing advancement in this research area is the identification of alkaliptosis, a novel form of cell death induced by the selective inhibitor JTC801, which triggers intracellular alkalization by blocking opioid-related opiate receptor-like 1 (Song et al., 2018b). Alkaliptosis exhibits biochemical and genetic characteristics distinct from those associated with other regulated cell death forms. For example, alkaliptosis lacks key features such as necrotic body assembly and lipid peroxidation. Furthermore, studies have shown that the inhibition of known cell death pathways fails to prevent alkaliptosis, underscoring its unique nature (Liu et al., 2020a; Song et al., 2018b).

**Figure 5.**
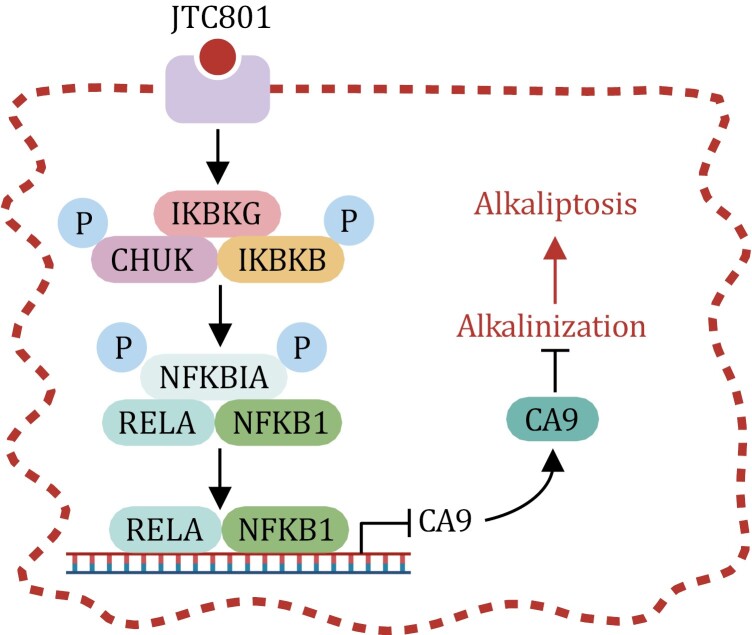
**Alkaliptosis regulatory mechanisms.** The canonical NF-κB pathway activation leads to the recruitment and activation of the IKK protein complex, comprising of CHUK, IKBKB, and IKBKG. Subsequently, the IKK protein complex phosphorylates and degrades NFKBIA, resulting in the nuclear translocation of NFKB1 or RELA to regulate target gene transcription. Alkaliptosis is driven by intracellular alkalinization upon JTC801 treatment, which promotes the transcription repression of *CA9* by NF-κB.

Mechanistically, the induction of alkaliptosis by JTC801 involves NF-κB activity, with blocking of the NF-κB pathway leading to a reduction in alkaliptosis (Song et al., 2018b) (Fig. 5). A key regulator of intracellular pH, carbonic anhydrase 9 (CA9), is implicated in this process because it governs the reversible hydration of carbon dioxide to bicarbonate (Swietach et al., 2009). NF-κB activation suppresses CA9, promoting alkaliptosis (Song et al., 2018b) (Fig. 5).

Given the common dysregulation of pH in cancer, targeting pH control has emerged as a rational strategy in cancer treatment (White et al., 2017). Recent findings have identified alkaliptosis as a promising approach for the treatment of diverse cancer types (Liu et al., 2020a). For instance, JTC801 displayed efficacy in suppressing tumor growth in a pancreatic cancer mouse model, without apparent toxicity (Song et al., 2018b). Additionally, JTC801-induced alkaliptosis showed potential in inhibiting the growth of venetoclax-resistant acute myeloid leukemia cells (Zhu et al., 2021). The selective toxicity of JTC801 against cancer cells might arise from differences between cancer cells and normal cells in the expression levels of pH-regulating molecules (Liu et al., 2020a). Alkaliptosis, therefore, harnesses the unique vulnerabilities of cancer cells driven by pH dysregulation, and it holds promise as a novel therapeutic strategy.

## Conclusions and perspectives

The exploration of metabolic cell death mechanisms in cancer has revealed a fascinating landscape of interconnected pathways, with each mechanism holding a unique potential for therapeutic intervention. While the discussions in this review have shed light on various metabolic cell death modes, they also highlight several unanswered important questions that require further exploration.

All of the cell death mechanisms presented in this review, with the exception of ferroptosis, have been identified in recent years, and their underlying mechanisms still remain largely unexplored. Pore-forming proteins, such as BAX and BAK in apoptosis, mixed-lineage kinase domain-like protein (MLKL) in necroptosis, and gasdermin D in pyroptosis, play pivotal roles in executing cell suicide programs (Vandenabeele et al., 2023). Intriguingly, the execution of cell sabotage pathways discussed in this review does not appear to involve such pore-forming proteins (however, it is essential to note that further in-depth investigations are warranted to address this important question); instead, the common feature among these cell death pathways is their induction by imbalances in cellular metabolism resulting from the depletion or overload of various metals or nutrients, underscoring the intricate interplay between cellular metabolism and the regulation of diverse cell death modalities. Future studies might uncover additional mechanistic similarities and differences among these cell sabotage pathways.

**Figure 6.**
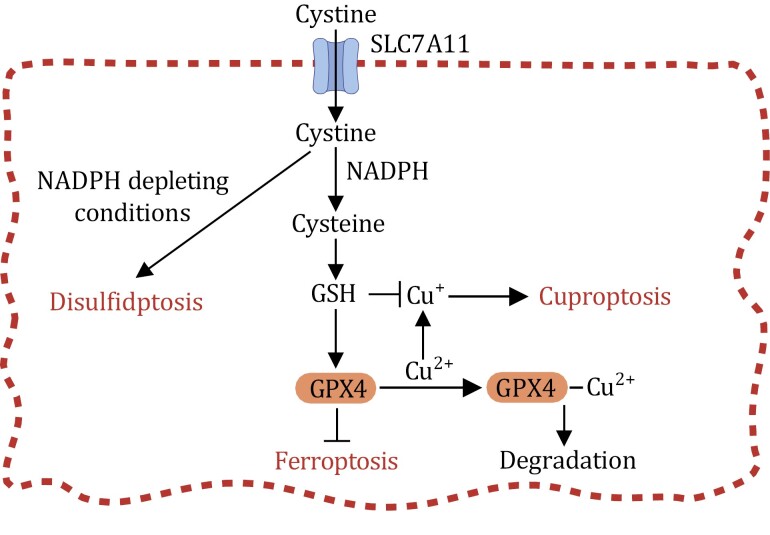
**Interplays among ferroptosis, disulfidptosis, and cuproptosis.** SLC7A11 facilitates the import of cystine into the cytosol. Cytosolic cystine serves as a critical precursor for synthesizing glutathione (GSH), a co-factor essential for GPX4-mediated suppression of ferroptosis. However, under conditions of NADPH depletion, an excessive buildup of cytosolic cystine can induce disulfidptosis. GSH also functions as an intracellular copper chelator, thereby suppressing cuproptosis. Additionally, copper can promote ferroptosis by instigating the autophagic degradation of GPX4.

Another important area for future research is to investigate the interplays among these cell death mechanisms. As discussed earlier, the interplay between ferroptosis and disulfidptosis is exemplified by SLC7A11-mediated cystine transport, wherein cystine transport suppresses ferroptosis yet promotes disulfidptosis (Fig. 6). Recent studies have shed light on additional crosstalk, particularly between ferroptosis and cuproptosis. For example, GSH, the critical co-factor for GPX4-mediated ferroptosis defense, also acts as an intracellular copper chelator, thereby mitigating cuproptosis (Fig. 6); consequently, GSH depletion by buthionine sulfoximine has been demonstrated to sensitize cancer cells to cuproptosis (Tsvetkov et al., 2022). Additionally, a recent study showed that copper promotes ferroptosis through inducing autophagic degradation of GPX4 (Fig. 6), with copper chelators, in turn, exhibiting the ability to suppress ferroptosis. These studies together highlight the intricate interplays among ferroptosis, disulfidptosis, and cuproptosis. Given the relatively recent discovery of most of these cell death pathways, it is anticipated that further exploration will unveil additional layers of crosstalk and interdependence among them. A comprehensive understanding of these dynamic interactions could provide fresh insights for novel therapeutic interventions.

From a translational standpoint, a formidable challenge in cancer therapy lies in achieving the selective destruction of cancer cells while sparing normal cells and immune systems. Although the compounds discussed in this review have exhibited efficacy in cancer cell lines and/or preclinical models, and although these cell death modes have revealed metabolic susceptibilities in specific cancers (e.g., disulfidptosis in SLC7A11-high cancers), the translation of these findings into meaningful therapeutic treatments for cancer patients demands comprehensive preclinical and clinical examinations.

Another significant challenge in translational research in these domains is the absence of established biomarkers that can effectively quantify the various metabolic cell death modes within cell lines or tissues. From our perspective, this challenge is rooted in the inherent mechanistic differences between cell suicide and cell sabotage programs (Green and Victor, 2012). Cell suicide programs are orchestrated by intricate signaling cascades and executed by specific cell death proteins (as exemplified by the pore-forming proteins discussed above), allowing researchers to gain a detailed mechanistic comprehension of the programs and the distinct protein modifications uniquely linked to each cell death pathway. This, in turn, facilitates the utilization of these modifications as reliable biomarkers for tracking their respective cell death processes—such as caspase-3 cleavage for apoptosis and MLKL phosphorylation for necroptosis.

In contrast, cell sabotage programs operate as metabolite-centric modes of cell death. The underlying metabolic alterations, such as lipid peroxidation in ferroptosis and disulfide accumulation in disulfidptosis, are intricately linked to a myriad of metabolic cues and can exert wide-ranging downstream effects. This intricate interconnectedness renders it more challenging to harness these metabolic alterations as biomarkers for accurately monitoring the corresponding cell death pathways. Nevertheless, delving deeper into the underlying mechanisms of these cell death pathways may yield fresh insights and uncover novel biomarkers that could ultimately lead to the development of effective therapeutic strategies to combat cancer.
